# Clinicopathological Features and Treatment of Ectopic Varices with Portal Hypertension

**DOI:** 10.4061/2011/960720

**Published:** 2011-07-31

**Authors:** Takahiro Sato, Jun Akaike, Jouji Toyota, Yoshiyasu Karino, Takumi Ohmura

**Affiliations:** Department of Gastroenterology, Sapporo Kosei General Hospital, Kita 3 Higashi 8, Chuo-ku, Sapporo 060-0033, Japan

## Abstract

Bleeding from ectopic varices, which is rare in patients with portal hypertension, is generally massive and life-threatening. Forty-three patients were hospitalized in our ward for gastrointestinal bleeding from ectopic varices. The frequency of ectopic varices was 43/1218 (3.5%) among portal hypertensive patients in our ward. The locations of the ectopic varices were rectal in thirty-two, duodenal in three, intestinal in two, vesical in three, stomal in one, and colonic in two patients. Endoscopic or interventional radiologic treatment was performed successfully for ectopic varices. Hemorrhage from ectopic varices should be kept in mind in patients with portal hypertension presenting with lower gastrointestinal bleeding.

## 1. Introduction

Portal hypertension can result in either the reopening of collapsed embryonic channels or reversal of the flow within existing adult veins [1]. Whilst esophagogastric varices are the most common complication in patients with portal hypertension, ectopic varices defined by large portosystemic venous collaterals occurring anywhere in the gastrointestinal tract, other than the esophagogastric region, are less common and account for between 1% and 5% of all variceal bleeding [2, 3]. Ectopic varices that are not esophagogastric are located predominantly in the duodenum, jejunum, ileum, colon, rectum, and enterostomy stoma. Bleeding from ectopic varices, which is rare in patients with portal hypertension, is generally massive and life-threatening. However, there are few reports on the clinicopathological features of ectopic varices. Endoscopic injection sclerotherapy (EIS) is now a standard procedure for the treatment of esophageal varices [4] and, recently, endoscopic band ligation (EBL) has been used widely to treat esophageal varices [5]. Balloon-occluded retrograde transvenous obliteration (B-RTO) is a new interventional modality for gastric fundic varices [6]. However, a definitive treatment has not been established for bleeding ectopic varices. 

 In this paper, we evaluate the clinicopathological features and treatment of ectopic varices in our ward.

## 2. Clinicopathological Features of Ectopic Varices

Esophagogastric varices are considered to be the most common complication in patients with portal hypertension, while ectopic varices (i.e., those outside the esophago-gastric region) are less common. Ectopic varices have been reported to occur at numerous sites, including 18% in the jejunum or ileum, 17% in the duodenum, 14% in the colon, 8% in the rectum, and 9% in the peritoneum [7]. 

From January 1994 to March 2009, we performed endoscopic or interventional radiologic treatment for 1218 portal hypertensive patients with esophagogastric varices. During this period, 43 patients were hospitalized in our ward for gastrointestinal bleeding from ectopic varices. There were 21 males and 22 females, ranging in age from 38 to 84 years (mean, 67.0 years). The underlying pathology of portal hypertension included liver cirrhosis (LC) in 22 patients, cirrhosis associated with hepatocellular carcinoma (HCC) in 9, primary biliary cirrhosis (PBC) in 3, idiopathic portal hypertension (IPH) in 6, extrahepatic portal vein obstruction (EHO) in 2, and another disease in 1 (Table 1). In terms of the clinical staging of cirrhosis, 22 patients were graded Child-Pugh class A, 18 class B, and 3 class C. The etiologies of LC were hepatitis B surface antigen (HBsAg) positivity in 4 patients, antibody to hepatitis C virus (anti-HCV) in 16 patients, alcoholic liver disease in 8 patients, sarcoidosis in 1 patient, and unknown in 2 patients. 

The frequency of ectopic varices was 43/1218 (3.5%) among portal hypertensive patients in our ward. The locations of the ectopic varices were rectal in 32, duodenal in 3, small intestinal in 2, vesical in 3, stomal in 1, and colonic in 2 patients (Table 2). Thirty-nine of 43 patients with ectopic varices had previously received emergency or prophylactic EIS for esophageal varices. Nine patients had a history of esophageal variceal bleeding, and emergency EIS had been performed in these cases. Prophylactic EIS had been performed on 30 patients with esophageal varices because of a high risk of bleeding.

## 3. Rectal Varices

Thirty two rectal variceal patients in our ward had had undergone EIS or EBL. There were 14 males and 18 females, ranging in age from 38 to 84 years (mean, 67.0 years). The underlying pathology causing portal hypertension included LC in 16 patients, cirrhosis associated with HCC in 7 patients, IPH in 4 patients, PBC in 3 patients, and EHO in 2 patients. In terms of the clinical staging of cirrhosis, 15 patients were graded Child-Pugh class A, 15 class B, and 2 class C. The etiologies of LC were HBs Ag-positivity in 3 patients, anti-HCV in 11 patients, alcoholic liver disease in 6 patients, sarcoidosis in 1 patient, and unknown in 2 patients. Thirty of 32 patients with rectal varices had previously received emergency or prophylactic EIS for esophageal varices, and esophageal varices were coexistent in two other patients.

Rectal varices represent portal systemic collaterals that are manifested as discrete dilated submucosal veins and constitute a pathway for portal venous flow between the superior rectal veins of the inferior mesenteric system and the middle inferior rectal veins of the iliac system. Rectal varices have been reported to occur at a high frequency in patients with hepatic abnormalities [8–10]. Massive bleeding from rectal varices occurs rarely, at a frequency ranging from 0.5% to 3.6% [11–13]. Rectal varices are an infrequent but potentially serious cause of hematochezia. 

Several diagnostic procedures have been performed to evaluate rectal varices, including endoscopy, magnetic resonance (MR) angiography, and endoscopic ultrasonography (EUS). Endoscopy is the principal method for diagnosis of rectal varices, and MR angiography is useful for evaluating the overall portosystemic collateral circulation [14]. EUS has become a useful modality for hemodynamic diagnosis of esophagogastric varices [15, 16]. The value of EUS [17–19] has been reported for the hemodynamic diagnosis of rectal varices, and Dhiman et al. found rectal varices via endoscopy in 43% and via EUS in 75%, of patients with portal hypertension [19]. Conventional EUS (7.5 or 12 MHz) reveals rectal varices as rounded, oval, or longitudinal echo-free structures in the submucosa and also shows perirectal veins outside the rectal wall [17–19]. EUS was considered superior to endoscopy or MR angiography in making a detailed diagnosis of rectal varices. Sato et al. demonstrated that intramural rectal varices, perirectal collateral veins, and the communicating veins between intramural rectal varices and perirectal collateral veins could be observed clearly via an ultrasonic microprobe [14]. 

Recently, percutaneous color Doppler ultrasonography (CDUS) has allowed us to detect the flow of blood in fine detail, and it has become widely accepted for the assessment of the hemodynamics of abdominal vascular systems, but few color Doppler findings of gastrointestinal varices have been reported. Komatsuda et al. reported the value of CDUS for the diagnosis of gastric and duodenal varices [20], and Sato et al. have reported the usefulness of CDUS for the hemodynamic evaluation of rectal varices [21]. 

CDUS cannot be performed successfully without a suitable acoustic window. Impediments such as bowel gas, body habitus, and cirrhosis limit the value of sonography for assessing the portal venous system. In addition, it is difficult to observe the collateral veins far from the probe with color Doppler sonography because of the limitations of Doppler sensitivity. The rectal wall was detected at the posterior area of the vagina in females and the prostate in males by sonography and rectal varices could be observed through the urine-filled bladder via CDUS. Sato et al. suggest that the measurement by CDUS of velocity in rectal varices is useful in diagnosing the grade of rectal varices. CDUS was very useful in screening for rectal varices in portal hypertensive patients [21]. 

Although EIS and EBL for esophageal varices are well-established therapies for esophageal varices, there is no standard treatment for rectal varices. Various medical treatments have been used to control bleeding from rectal varices, but none of these is currently considered to be a standard method. Surgical approaches include portosystemic shunting, ligation, and under-running suturing [8]. Some investigators have reported that interventional radiologic techniques such as transjugular intrahepatic portosystemic shunts (TIPSs) were successfully employed for rectal variceal bleeding [22–24]. Wang et al. first reported the usefulness of EIS in treating rectal varices and found it to be effective for controlling bleeding [25]. EBL was introduced as a new method for treating esophageal varices, and it is reportedly easier to perform and safer than EIS. Several cases of successful treatment of rectal varices using EBL have been reported [26–28]. Levine et al. treated rectal varices initially with EIS, and 1 week later, EBL was performed on the remaining rectal varices. These investigators described EBL as a safe and effective therapy for rectal varices. On the other hand, Sato et al. retrospectively evaluated the therapeutic effects and rates of recurrence of rectal varices after EIS or EBL [29], and EIS was successfully performed without complications. The recurrence rate did not differ significantly between the EIS and EBL groups, although recurrence tended to be more frequent with EBL. It is necessary to evaluate the hemodynamics of the rectal varices before EIS to avoid severe complications such as pulmonary embolism, and the sclerosant should be injected slowly under fluoroscopy, taking care to ensure that the agent does not flow into the systemic circulation. 

A standard therapy for rectal varices has not been established. More investigations are needed in larger numbers of patients before evidence-based treatment recommendations can be made. 

## 4. Duodenal Varices

Three duodenal variceal patients (2 males and 1 female) underwent interventional radiology in our ward. The underlying pathology causing portal hypertension included LC in two patients, and IPH in one. In terms of the clinical staging of cirrhosis, all three were graded Child-Pugh class A. The etiologies of LC were anti-HCV-positivity in one patient and alcoholic liver disease in the other. All three patients with duodenal varices had previously received emergency or prophylactic EIS for esophageal varices. The sites of the duodenal varices were the second portion of the duodenum in one case and the distal third portion in two.

The duodenum is a rare site of variceal hemorrhage in patients with portal hypertension but bleeding from duodenal varices is generally massive and life-threatening. It is seen not only in patients with extrahepatic portal hypertension but also in patients with cirrhosis of the liver [30–32]. Duodenal varices are considered to be ectopic varices and account for 1–3% of all varices in patients with liver cirrhosis [33]. Diagnosis of ruptured duodenal varices and control of bleeding are difficult. 

The duodenum can be a site of severe variceal hemorrhage, with mortality as high as 40% from the initial bleeding [34, 35]. Although more commonly associated with extrahepatic portal hypertension, duodenal varices may occur in intrahepatic portal hypertension. Cirrhosis of liver is the most common intrahepatic cause of duodenal varices, accounting for 30% of cases [31, 36]. Extrahepatic causes vary and include portal vein thrombosis and obstruction of the splenic vein and inferior vena cava [31, 37, 38]. 

The most common site of duodenal varices is the duodenal bulb [35], followed by the second portion of the duodenum [39]. Varices in the duodenal bulb, which occur most frequently in the United States and Europe, are caused by extrahepatic portal obstruction. In Japan, duodenal varices are observed more commonly in the second portion of the duodenum [40, 41]. On the other hand, duodenal varices in the distal third portion are very rare [42, 43]. Duodenal varices are formed by the developed collateral veins originating from the portal vein trunk or superior mesenteric vein, which empty into the inferior vena cava [31, 34].

The bulb and second portion of duodenum can be observed endoscopically. However, location of the bleeding site often is difficult in the duodenum. In our two cases of duodenal varices in the distal third portion, we could not observe the varices by fibergastroscopic examination and we suspected rupture of duodenal varices via computed tomography (CT). 

Duodenoscopy and double-balloon enteroscopy were very useful in evaluating the duodenal varices in the distal third portion. 

Recently, medical treatments with interventional radiology and endoscopic procedures have been reported for duodenal varices. EBL for bleeding duodenal varices is challenging because of the difficulty in maintaining the field of vision. EBL may be useful for temporary hemostasis [40, 41] but rebleeding of duodenal varices is a problem with EBL. Additional treatment is recommended following EBL for duodenal varices. EIS has been reported to be successful in controlling duodenal variceal bleeding [44, 45] but there have been reports of cases of rebleeding of duodenal varices after EIS [35, 46]. N-butyl-2-cyanoacrylate (Histoacryl, B.Braun Dexon GmbH Spangenberg, Germany) is a tissue glue monomer that instantly polymerizes and solidifies upon contact with blood. Endoscopic obliterative therapy with Histoacryl seems to be a useful method for bleeding gastric varices [47, 48], and it is also suitable for emergency duodenal variceal bleeding [41, 42]. 

Interventional radiologic treatment options for duodenal varices include TIPS, B-RTO, and percutaneous transhepatic obliteration (PTO). B-RTO was successfully performed for two cases of duodenal varices, and PTO for one case, in our ward. Successful treatment of duodenal varices by TIPS [35] and B-RTO [49–51] has been reported. Although TIPS is a relatively safe and effective means of decompressing the portal pressure, it has a certain limitation in patients with severe liver atrophy and complications such as encephalopathy and cerebral embolization. B-RTO can obliterate not only varices but also the afferent and efferent veins and should be considered for treating duodenal varices. Successful treatment of duodenal varices by PTO has been reported [52, 53]. 

## 5. Small Intestinal Varices

Two small intestinal variceal patients (both male, one jejunal varices, and one ileal varices) had undergone interventional radiology in our ward. The underlying pathology causing portal hypertension was LC in both. In terms of the clinical staging of cirrhosis, one patient was graded Child-Pugh class A and the other class B. The etiologies of LC were HBs Ag-positivity in one patient and anti-HCV-positivity in the other. The jejunal variceal case had previously undergone gastropylorectomy, and esophageal varices coexisted. The ileal variceal patient had previously received prophylactic EIS for esophageal varices and surgery on the ileocecum to remove a benign colonic tumor. 

When repeat upper and lower endoscopies are negative in gastrointestinal bleeding, the small intestine should be investigated. Most bleeding jejunal and ileal varices, generally detected previous to intra-abdominal surgery, are serious because of the difficulty of early diagnosis. 

In our two cases, the patients' risk factors included portal hypertension due to liver cirrhosis, and previous surgery. Collaterals formation within adhesions from previous surgery is the usual mechanism for the development of ectopic varices [7]. Adhesions tend to bring the parietal surface of the viscera in contact with the abdominal wall, and portal hypertension results in the formation of varices below the intestinal mucosa. 

**Figure 1:**
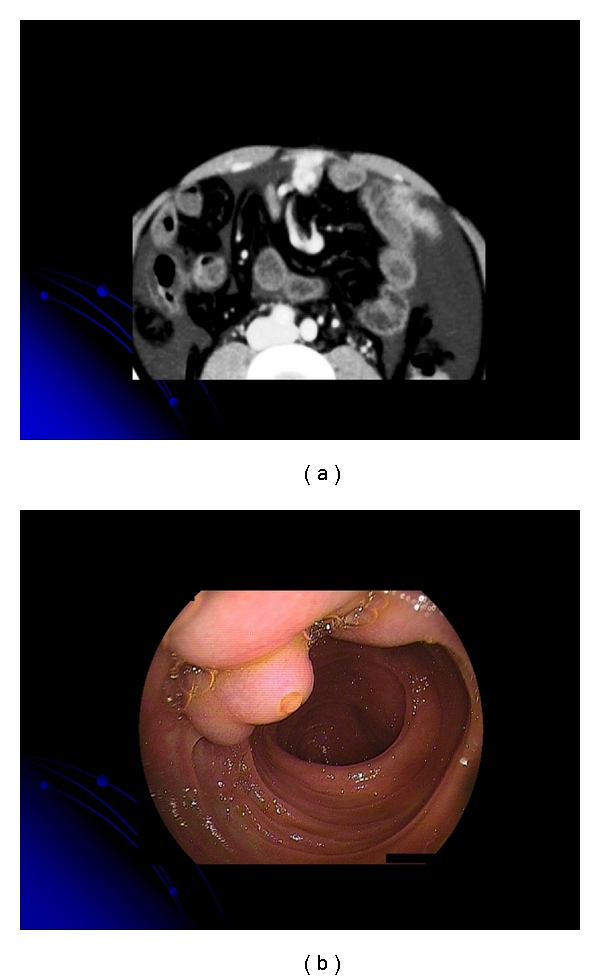
(a) Computed tomography showing the vessel image in the jejunum. (b) Double-balloon enteroscopy revealed jejunal varices with a white plug.

 Location of the bleeding site often is difficult in the intestinal varices. In our two cases, we suspected rupture of intestinal varices via CT. We show jejunal variceal varices, and CT and double-balloon enteroscopy were useful in evaluating the jejunal varices (Figures 1(a) and 1(b)). Lim et al. have reported the usefulness of capsule endoscopy for the diagnosis of bleeding jejunal varices [54]. 

**Figure 2:**
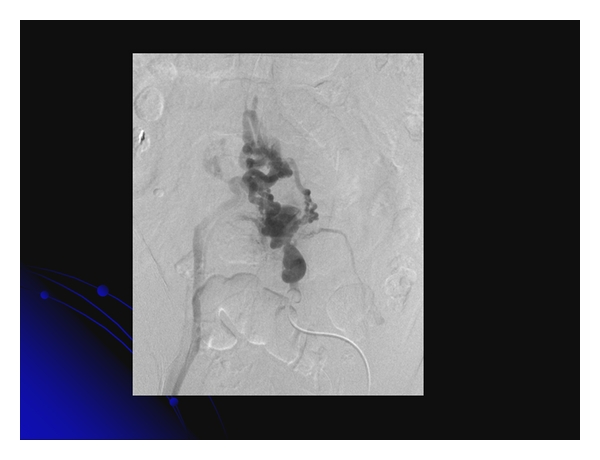
Balloon-occluded retrograde transvenous obliteration was performed successfully for jejunal varices.

 Several cases of bleeding jejunal [54–59] and ileal varices have been reported [60–68]. A triad of portal hypertension, hematochezia without hematemesis, and previous abdominal surgery characterizes small intestinal varices [69]. Several approaches for the treatment of jejunal varices include surgery [55], portal venous stenting [56, 58, 59], and percutaneous embolization [54, 57]. Surgical approaches such as segmental resection and ligation generally control bleeding from ileal varices successfully [64, 65, 70, 71]. In patients with a poor condition, interventional radiologic treatments, such as insertion of a TIPS for ileal varices, have been performed as a nonsurgical treatment option [3, 66, 68]. Because B-RTO can obliterate not only varices but also the afferent and efferent veins, it is practical for treating ileal varices [72], as described here. In the future, interventional radiologic treatments such as B-RTO may also be applied as therapy for patients in a poor condition. In our cases, B-RTO was successfully performed for jejunal (Figure 2) and ileal varices.

## 6. Vesical Varices

Because our three vesical variceal patients (2 males and 1 female) had a history of EIS for esophageal varices and two had received abdominal surgery, the usual collateral veins from portal hypertension may have been disrupted. Collateral formation within adhesions from previous surgery is the usual mechanism for the development of ectopic varices [7]. The underlying pathology causing portal hypertension was LC in one patient, LC-associated HCC in another, and IPH in the third. In terms of the clinical staging of cirrhosis, one patient was graded Child-Pugh class A, one class B, and one class C. The etiologies of LC were alcoholic in one patient and anti-HCV-positivity in the other. 

Bleeding from vesical varices is rare in patients with portal hypertension [7, 73–76] because the bladder wall is an unusual collateral route for the venous splanchnic blood. Previously reported cases of vesical varices had a history of abdominal surgery [73, 74, 76, 77], so that the vesical varices might have appeared after surgery that provided an unusual collateral route resulting from portal hypertension. 

**Figure 3:**
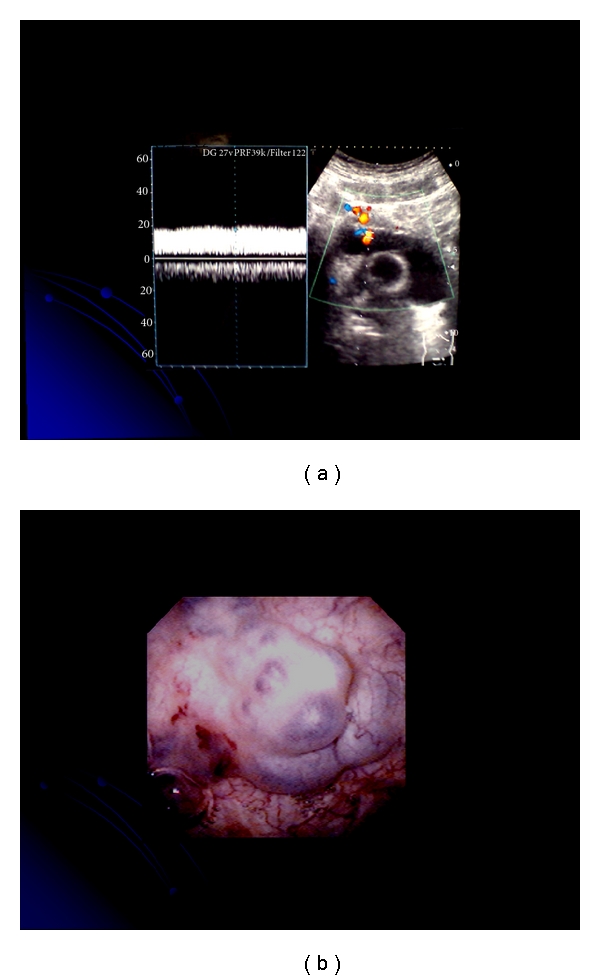
(a) Color flow images of vesical varices can be delineated clearly in the urine-filled bladder. (b) Cystoscopic examination revealed vesical varices on the anterior wall of the bladder.

 CDUS also is very useful for screening for collateral vessels in portal hypertensive patients, in that it can be performed repeatedly. Color flow images of vesical varices can be delineated clearly in the urine-filled bladder. CDUS is very useful for the diagnosis of vesical varices (Figure 3(a)), as is CT. Cystoscopic examination revealed vesical varices on the anterior wall of the bladder in our cases (Figure 3(b)). 

No definitive treatment has been established for bleeding vesical varices. We used PTP to reveal the detailed hemodynamics of the collateral circulation in vesical variceal patients, including the afferent and efferent veins, and these patients were successfully treated with PTO [77].

## 7. Colonic Varices

Two colonic variceal patients (both female, one descending colonic varices and one transverse colonic varices) underwent interventional radiology (PTO) and EIS in our ward. The underlying pathology causing portal hypertension was LC in both. In terms of the clinical staging of cirrhosis, both were graded Child-Pugh class A. Both were anti-HCV-positive and had a history of EIS for esophageal varices, and one had received abdominal surgery. 

The most common sites of colorectal varices are the rectum and cecum [78]. Colonic varices can be associated with several conditions, such as portal hypertension, portal venous obstruction, postsurgical changes, and idiopathic factors [3, 79–84]. 

Colonoscopy is the principal method for the diagnosis of colonic varices, and MR angiography is useful for evaluating the overall portosystemic collateral circulation. CT has been reported rarely but has shown a colonic wall which is thickened with a scalloped appearance [85]. In our cases, we suspected colonic varices via CT [86]. 

 Several therapies, including PTO, colonic resection, portacaval shunt construction, endoscopic procedures, TIPS, variceal embolization, and B-RTO, have been reported [3, 79–84, 86–90]. The treatment of colonic varices is not well defined. 

## 8. Stomal Varices

The (male) stomal variceal patient in our ward had undergone interventional radiology (TIPS). The underlying pathology causing portal hypertension was LC (Child-Pugh class B), and he was anti-HCV-positive. This case had previously received Miles' operation for rectal cancer, and EIS had been performed for esophageal varices.

Stomal varices can occur in patients with stoma in the presence of portal hypertension and remain difficult to diagnose and manage. The overall morbidity of the stomal varices is much higher given the propensity for recurrence and massive bleeding, requiring multiple blood transfusions [91, 92]. 

The mechanism of stomal variceal hemorrhage is related to variceal erosion or local trauma. Several management strategies have been described for stomal variceal hemorrhage, including local therapy, EIS, TIPS, B-RTO, and surgery. Although local therapies are effective for the initial control of bleeding, these may be not effective in preventing recurrent bleeding [93]. EIS is effective for controlling stomal variceal bleeding [94], and portosystemic surgery is effective for prevention of recurrent bleeding but also is associated with significant morbidity and mortality [95]. PTO has been used safely for acute stomal variceal bleeding [92, 96, 97]; however, recurrent bleeding is frequent. TIPS is an effective therapy for bleeding stomal varices [98–100] but may result in a higher mortality of patients with severe decompensated liver function because of encephalopathy, rather than the stomal variceal bleeding itself [96]. Recently, Minami et al. have reported that B-RTO was useful for recurrent hemorrhage from stomal varices [101].

## 9. Conclusions

It is difficult to determine the best treatment strategy for ectopic varices because of inaccessibility, initial difficulty in diagnosis, and subsequent difficulty in treatment. Hemorrhage from ectopic varices should be kept in mind in patients with portal hypertension presenting with lower gastrointestinal bleeding.

## Figures and Tables

**Table 1:** Underlying pathologies in patients with ectopic varices.

	Cases (*N*)
Liver cirrhosis	22
Cirrhosis associated hepatocellular carcinoma	9
Idiopathic portal hypertension	6
Primary biliary cirrhosis	3
Extrahepatic portal vein obstruction	2
Other	1

Total	43

**Table 2:** Sites of ectopic varices (*n* = 43).

Site	Cases (*N*)
Rectal varices	32
Duodenal varices	3
Small intestinal varices	2
Vesical varices	3
Colonic varices	2
Stoma varices	1

Total	43
